# Prevalence of insomnia and poor sleep quality in the prison population: A systematic review

**DOI:** 10.1111/jsr.13677

**Published:** 2022-07-05

**Authors:** Nia Sheppard, Lee Hogan

**Affiliations:** ^1^ North Wales Clinical Psychology Programme Bangor University Bangor UK; ^2^ Betsi Cadwaladr University Health Board Bangor UK

**Keywords:** adult, insomnia, prevalence, prison, sleep, systematic review

## Abstract

Insomnia is a public health concern with several associated negative health‐related outcomes. Risk factors for insomnia place the prison population at an increased risk of inadequate sleep. This paper systematically reviewed the literature reporting on the prevalence of insomnia and poor sleep quality in the prison population. Following a comprehensive database search and screening process, 12 studies were eligible for inclusion in this review. Six studies provided prevalence rates for insomnia and nine for poor sleep quality. Varied prevalence rates were found for insomnia (i.e. 26.2%–72.5%) and poor sleep quality (i.e. 42.8%–88.2%). Evaluation of the prevalence rates revealed varied measurement of sleep quality, inconsistent parameters of standardised measures, and methodological heterogeneity. Other considerations were given to geographical differences, the use of self‐report data, gender difference, environmental factors and comorbidities of insomnia. The review highlighted an increase in the quantity of studies and some improved quality; although the findings were highly variable, in the main, inadequate sleep amongst prisoners was higher than the general population. Limitations of the review and implications for further research are discussed.

## 1 INTRODUCTION

Insomnia is defined as difficulty initiating or maintaining sleep, which results in sleep dissatisfaction and daytime impairment (American Academy of Sleep Medicine, 2014). Population prevalence rates reported in a variety of studies globally, estimated that approximately 30% of adults report one or more insomnia symptom (Roth, 2007). To formally diagnose insomnia disorder, the Diagnostic and Statistical Manual of Mental Disorders, 5th edition (DSM‐5) specifies one of three symptoms: (1) difficulty initiating sleep; (2) difficulty maintaining sleep (frequent awakenings); or (3) unwanted early‐morning awakenings (with inability to return to sleep). The symptoms must cause distress or impairment in functioning, be present for at least three times a week for 3 months, and not be attributable to another sleep disorder, psychiatric disorder or drug usage (American Psychiatric Association, 2013). In accordance with this diagnostic criteria, the population prevalence (in USA and Europe) is believed to be about 6%–10% (Morin & Benca, 2012; Riemann et al., 2017; Roth, 2007). Geographical variations have been found, including lower insomnia prevalence in Asian countries (Cao et al., 2017).

Due to the subjective and personal nature of sleep, diagnosing insomnia can be difficult; additionally, diagnostic criteria continue to evolve as we find out more about insomnia (National Sleep Foundation, 2020a). There are many standardised measurements designed to identify insomnia symptoms, such as the Insomnia Severity Index (ISI; Bastien, 2001) and the Pittsburgh Sleep Quality Index (PSQI; Buysse et al., 1989), alongside diagnostic interview, sleep diary and objective sleep measurement (e.g. polysomnography and actigraphy). These types of measures capture clinical and subclinical insomnia, or poor‐quality sleep. There are a number of risk factors known for insomnia: for example, being female is a risk factor for insomnia, and the risk of insomnia also increases with age; insomnia is often comorbid with mental health conditions, substance misuse and certain medical complaints (Bos & Macedo, 2019; Morin & Benca, 2012; National Sleep Foundation, 2020a).

Prisoners are a vulnerable group who have an elevated risk of insomnia. The prison environment can have a negative impact on sleep for a large proportion of inmates due to poor sleep hygiene because of institutionalisation, boredom, noise, overcrowding, fear, lack of autonomy, substance misuse, light, temperature and discomfort (Barker et al., 2016; Dewa et al., 2015, Dewa et al., 2017a; Elger, 2007). In addition, prison populations are known to have elevated incidences of mental health conditions (Fazel & Danesh, 2002), consequently increasing the risk further (Dewa et al., 2015).

It is important that there is appropriate recognition and management of insomnia in prisons. The consequences of substandard sleep include cognitive impairment, increased aggression, reduced impulse control, emotional dysregulation, increased risk of accidents and serious physical health conditions directly linked to mortality (for review, see Bos & Macedo, 2019). The National Sleep Foundation (2020b) recommends that adults have between 7 and 9 hr of sleep a night to help mitigate such risks.

There have been two notable reviews previously: Elger (2007) conducted a non‐systematic scoping review into insomnia in prison settings that comprised nine studies; and more recently, Dewa et al. (2015) conducted a comprehensive integrative review looking into prevalence, correlates and management of insomnia in prisons. Dewa et al. systematically reviewed 33 studies and identified five themes: (1) the varied prevalence of insomnia; (2) the comorbidity with psychiatric disorder and substance misuse; (3) the negative impact of environmental factors in prisons; (4) the prescription of hypnotic medication; and (5) evidence that non‐pharmacological treatment can help improve sleep. Twelve of the studies reviewed reported the prevalence of insomnia, where prevalence was found to range from 10.9% to 81% based on studies between 1974 and 2012. They concluded that the reviewed studies varied significantly in their quality and in the measurement of insomnia (e.g. a lack of standardised tools matched to diagnostic criteria and of insomnia measured as an impartial factor rather than within the measurement of comorbid conditions). Included within the recommendations were calls for future research to: (a) use validated and objective measures of insomnia where possible; (b) improve the estimation of prison insomnia; and (c) develop protocols to intervene appropriately.

Since the completion of the current review, a further relevant review has been published. Griffiths and Hina (2022) conducted a review of insomnia interventions in prisons, which included a secondary analysis of prevalence of insomnia. The search timeframe was within the one used in this review, and they had broader inclusion criteria. They concluded that the prevalence of insomnia in prisoners is high across the world.

The primary aim of this paper was to systematically review the literature on insomnia and poor sleep quality prevalence in prison populations since the previous review, and to examine whether there had been improvement in the quantity and quality of the research. It was hoped that this would provide a more accurate estimation of prevalence, which would help support the importance of recognition and subsequent management of insomnia and poor sleep quality in prisons.

## 2 METHODS

### 2.1 Search strategy

Electronic databases were searched in February 2021. The databases searched were Web of Science (core collection by “topic”); CINAHL (including MEDLINE, in unselected fields by Boolean search mode); PsycINFO (in “anywhere”) and PubMed central (in all fields). Restrictions placed upon the search criteria were publication dates between 2010 and 2021 (i.e. to reduce duplication of studies included in previous review), full‐text, English language and peer‐reviewed publications. The search terms used were: (“sleep quality” OR “sleep disorder*” OR “sleep disturb*” OR “disturbed sleep*” OR “poor sleep” OR insomnia* OR sleepiness OR sleepless* OR “sleep duration” OR “sleep problem*” OR “sleep hygiene” OR circadian* OR nightmare* OR “sleep deprivation”) AND (prison OR prisoner OR imprison* OR inmate OR correctional OR jail OR custody OR offender OR detainee OR incarcerat*).

### 2.2 Study selection

**FIGURE 1:**
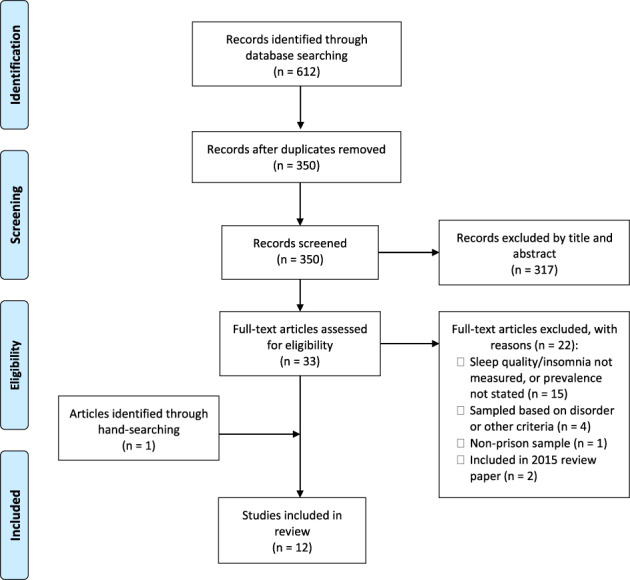
PRISMA flow diagram of literature search and screening

Following PRISMA guidelines (Moher et al., 2009), the study selection process is depicted in Figure 1. The search returned 350 unique articles, once 262 duplicates were removed. Studies were screened by title and abstract, which removed 317 articles. Full texts were retrieved for the remaining 33 articles where the following inclusion/exclusion criteria were applied: (1) contained an adult prison sample of participants (male and/or female); (2) measured sleep quality or insomnia (i.e. standardised questionnaire such as PSQI or ISI, unstandardised questionnaire, interview or actigraphy); (3) were an unselected general sample (i.e. not groups limited to certain characteristics, for example by age or psychiatric disorder); and (4) reported prevalence rates of sleep quality or insomnia. Additionally, studies included in an earlier review (Dewa et al., 2015) were excluded so that this would be an updated review of prevalence of sleep problem within the prison population. A further 22 studies were excluded based on these criteria. Hand‐searching of the reference lists and citations of the included studies revealed one additional study that met inclusion criteria.

All initial screening was conducted by the first author in the interest of time due to COVID‐19 restrictions. Full texts were assessed for eligibility by the first author; 10% (of the 33 articles) were randomly selected (using Excel) and counter‐reviewed independently by the second author. There was full agreement on papers included and excluded with no disputes.

### 2.3 Data extraction and analysis

A data extraction table was designed by both authors based on the previous review, and the critical appraisal criteria was used to extract the relevant data from the 12 studies in this review. The extracted data included authors, year of publication, location of study, study design, sample size, participant age and gender, details of the prison setting, sampling method (including any exclusion/inclusion criteria), measure of sleep used and its reliability, aim of the study and sleep‐related outcomes, and prevalence rate(s).

Assessment of study quality was undertaken during data extraction using the standardised critical appraisal checklist for studies reporting prevalence data (Joanna Briggs Institute, 2014; Munn et al., 2015). Given that there is no single, standardised critical assessment tool for all study designs, a different tool to that used by Dewa et al. (2015) was used given the differing scope and research design of their review. Studies were assessed according to sampling (size and approach), response rate, study objectives, data analysis, identification of insomnia and measurement. The quality assessment utilised a standardised form (Munn et al., 2015) and each of the nine domains was given a score; total scores were used to evaluate study quality (7–9 “good” and 4–6 “fair”). Quality assessment and data extraction was completed by the first author and reviewed by the second author at various stages throughout data extraction, with full agreement. Risk of bias was minimised by using the quality assessment tool and adhering to PRIMA guidelines.

Due to the heterogeneity in the method of measurement of sleep quality/insomnia, it was not appropriate to conduct a meta‐analytic estimation of prevalence. A descriptive, narrative approach was used to summarise the key findings.

## 3 RESULTS

### 3.1 Overview

The literature search identified 12 cross‐sectional studies that met the inclusion criteria. Table 1 presents data extracted from each of the 12 studies (one article, see Barker et al., 2016 details two unique studies and is described over two rows). The table begins with studies measuring insomnia over the first six rows in ascending publication date order, followed by those measuring sleep quality. Overall, using Munn et al.’s (2015) appraisal checklist, the quality of studies was adequate, especially when comparing with Dewa et al.’s (2015) previous review where the quality of reviewed studies varied considerably. Eight studies were rated as “good” and four were rated as “fair” (Table 1). Most studies utilised standardised measures of sleep quality/insomnia (*n* = 10) and had good response rates, with one‐third reporting sample size calculations. Generally, studies with quality rated as “fair” lacked detail with regards to the setting and sampling methods.

**TABLE 1:** Summary of key findings and prevalence rates of studies included in review

Author, year, country and quality rating	Study design	Participants	Setting and sample	Sleep measure and reliability	Main aim and sleep‐related results	Prevalence
Vogler et al. (2014) Switzerland Good quality	Cross‐sectional Self‐completed questionnaire	*N* = 49 males Aged 21–73 years (mean 39.37 years, SD 13.95)	Open prison, all male (106 total capacity at time of study) All prisoners screened, 32 excluded based on, language ability, meeting criteria for ICD‐10 “mental retardation” or had legal guardian (66% response rate); 28 (57%) general prison section, 18 (37%) privileged section	Standardised: ISI (translated to German) Reliability α = 0.85 Cut‐off score 10 used for subclinical insomnia Unstandardised: sleep hygiene and sleep duration	Investigation of sleep in relation to anger, ADHD, depression, physical health and life satisfaction Short and poor sleep was related to anger and more physical health complaints Poor sleep also related to more rumination and symptoms of ADHD No sig. Difference in sleep between prison sections	33% subclinical insomnia/poor sleep quality (ISI ≥ 10) 37% slept < 6 hr a night
Dewa et al. (2017a) UK Good quality	Cross‐sectional Questionnaire completed via interview	*N* = 237 (118 male, 119 female) Aged 18–72 years (mean 36.2 years, SD 11.9)	Two adult male prisons (category B and category C), and one prison for adult females Total capacity of 3 prisons 2186 at time of study Random sampling Excluded those unable to provide informed consent, risks that prevented being interviewed by a lone professional and not English speaking (mean response rate 64%) Sample size calculated	Standardised: SCI Reliability α = 0.89 Score ≤ 16 possible insomnia PSQI Reliability α = 0.89 Score ≥5 indicated poor sleepers SHI Reliability r = 0.71 DBAS‐16 Reliability α = 0.79	Study of prevalence of insomnia and associated forensic risk factors in prisoners in England. Higher prevalence of insomnia in prison than in UK general population (using the same measure, SCI) Prisoners with insomnia sig. more likely to have symptoms of anxiety, depression, suicidality and suspiciousness	61.6% insomnia (DSM‐5 criteria using SCI) 70.6% females 52.5% males 88.2% poor sleep quality (PSQI > 5)
Raha et al. (2018) India Good quality	Cross‐sectional Questionnaire completed via interview	*N* = 80 (40 male, 40 female) Mean age 36.74 years (SD 10)	Central jail Consecutive sampling, matched for gender at intake Excluded based on language barriers, diagnosed psychiatric illness, hearing or vision impairments or those with serious medical illness Sample size calculated	Standardised: PIRS (also translated to Assamese and Bengali) Reliability stated as “good”	To compare the prevalence of insomnia, depression and suicidality between male and female inmates Higher prevalence in female inmates in all defined problems	72.5% insomnia in females 65% insomnia in males
Li and Lai (2019) Taiwan Good quality	Cross‐sectional Self‐completed questionnaire	*N* = 1490 males Aged 21–77 years (mean 44.73 years, SD 10.01)	Prison, total capacity 1595 at time of study (8 female) All prisoners screened ‐ excluded pre‐trial, those under observation or rehabilitation, females and juveniles (97.99% response rate)	Standardised: ISI‐C (Chinese version) Reliability α = 0.94 Cut‐off score 9 used for insomnia	Study of prevalence and correlates of insomnia in Taiwan prisoners Insomnia prevalence in prison was higher than adult community population Insomnia was independently related to religious beliefs, anxiety and self‐rated health status	26.9% insomnia (ISI ≥ 9)
Acar et al. (2019) Turkey Fair quality	Cross‐sectional Questionnaire completed via interview	*N* = 399 (389 male, 10 female) Age 18–75 years (mean 34.54 years, SD 9.93)	Type M prison (Turkey) male and female All prisoners screened Excluded if less than 1 month of incarceration	Standardised: ISI (Turkish version) Reliability α = 0.79 Cut‐off score ≥ 15 PSQI (Turkish version) Reliability not reported Score ≥ 5 indicated poor sleepers MEQ (Turkish version) Reliability α = 0.81	To explore whether PTSD dissociative subtype differentiated with regard to sleep disturbance, circadian preference and posttraumatic cognitions Insomnia and poor sleep quality were found to be pronounced among prisoners with PTSD irrespective of levels of dissociation	37.8% clinical insomnia (ISI ≥ 15) 70.4% poor sleep quality (PSQI ≥ 5)
Geng et al. (2020) China Good quality	Cross‐sectional Questionnaire completed via interview	*N* = 1491 males Age 18–69 years (mean 35.44 years, SD 9.67)	Maximum security men's prison (total capacity 2358 at time of study) Voluntary sampling Excluded based on sight problems, not completing primary school education, those under observation and serious mental illness (87.3% response rate of 1708 who met criteria)	Unstandardised questionnaire: 6 items to evaluate sleep duration, initiation, maintenance, early awakening, daytime dysfunction and quality on a four‐point scale (except duration) Insomnia classed in accordance with DSM‐5 and ICSD‐3 as daytime dysfunction plus initiation, maintenance or early awakening issues	To investigate the prevalence and correlates of sleep problems in prison in China Poor physical health, PTSD and depression were associated with insomnia and poor sleep quality Prevalence of insomnia was nearly two times higher than in general Chinese population	26.2% insomnia symptoms 45.9% poor sleep quality 17.4% slept < 6 hr a night
Harner and Budescu (2014) USA Good quality	Cross‐sectional Self‐completed questionnaire	*N* = 438 females Age 20–85 years (mean 38.29 years, SD 10.93)	Maximum security women's prison (total capacity 1549 at time of study) Voluntary sampling Only included inmates of the prison's “general population” (48% response rate of 900 invited)	Standardised: PSQI Reliability α = 0.81 Scores ≥ 6 indicated poor sleepers Reliability α = 0.70)	To describe incarcerated women's sleep quality and associates including risk for sleep apnea Poor sleepers scored significantly higher on the risk for sleep apnea scale compared with women who did not meet the poor sleep threshold Risk for sleep apnea was low overall	72% poor sleep quality (PSQI > 5)
Barker et al. (2016) (study 1 of 2 reported) UK Fair quality	Cross‐sectional Self‐completed questionnaire	*N* = 95 males Mean age 35.25 years (SD 10.9)	UK adult male prison All prisoners at the prison at time of study approached (37.6% response rate of 300 invited)	Standardised: PSQI Reliability not stated Cut‐off used not stated PSQI question 9 used for analysis measuring *perceived* quality of sleep as good (fairly or very) or bad (fairly or very)	Study 1 explored explicit aggression and implicit processing in relation to sleep quality and quantity Sleep quantity and quality did not associate with aggression, but those *perceiving* poor sleep quality were more likely to report higher levels of aggression	56% *perceived* poor sleep quality Mean PSQI 9.07 (SD 4.4) 40% slept < 6 hr a night
As above, study 2 of 2 reported	Cross‐sectional Self‐completed questionnaire	*N* = 141 males Mean age 19.15 (SD 1.24)	UK young adult male prison All prisoners at the prison at time of study approached (18% response rate of 500 invited)	As above	Study 2 extended the aggression variables to address hostile attribution, prosocial attribution and aggression motivation Poor sleep quality was associated with lower prosocial attribution tendencies and higher aggression Those *perceiving* poor sleep were also more likely to report higher levels of aggression	61.7% *perceived* poor sleep quality Mean PSQI 9.80 (SD 4.1) 36.9% slept < 6 hr a night
Goudard et al. (2017) France Fair quality	Cross‐sectional Self‐completed questionnaire	*N* = 358 (319 male, 39 female) Age not stated	Total capacity of prison 690 adults (60 for females) All prisoners invited Excluded recent arrivals (56% response rate of 664 invited)	Unstandardised: bespoke questionnaire relating to sleep satisfaction, duration, sleep hygiene, insomnia symptoms and hypnotic treatment	To define the therapeutic profile of inmates treated for insomnia Most common symptom of insomnia was several awakenings at night, and most frequent reported aetiologies were rumination and noise Most reported that sleeping problems began/worsened after incarceration A quarter of inmates were taking hypnotic treatment, and most began treatment in prison	56% dissatisfied with sleep (21% “bad” 35% “quite bad”) Approx. 35% slept < 5 hr a night
Ishfaq and Kamal (2019) Pakistan Fair quality	Cross‐sectional Questionnaire self‐completed or with assistance from literacy teacher	*N* = 362 (349 male, 13 female) Age 19–70 years (mean 34.9 years, SD 10.11)	Two central prisons Sampling not stated 182 prisoners from one prison and 180 from the other	Standardised: DSM‐5 CCSM (translated to Urdu) Reliability α = 0.89 1 item for sleep disturbance (23 total items) using a five‐point rating scale Scores ≥ 2 indicate clinically relevant symptom	To translate CCSM and measure comorbid psychiatric symptomology among prisoners Sleep problem was the most reported symptom at the severe end of the scale (score 4, reported on a daily basis)	42.8% sleep problems
Getachew et al. (2020) Ethiopia Good quality	Cross‐sectional Questionnaire overseen by data collectors	*N* = 421 (393 male, 28 female) Age 18–72 years (mean 31.35 years, SD 10.33)	Contained prisoners who were sentenced and awaiting court decision (total capacity 1500 at time of study) Random sampling Excluded those awaiting court decision, with diagnosed psychiatric disorder, those in isolation and with chronic physical illness (99.5% response rate of 423 invited) Sample size calculated	Standardised: PSQI Reliability not stated Score >5 indicated poor sleepers SHI Reliability stated as “acceptable”	To determine the prevalence of poor sleep quality and associated factors among prisoners More than half of the participants had poor sleep quality Depression, poor sleep hygiene and certain crime types were associated with poor sleep	62.5% poor sleep quality (PSQI > 5)
Abdu and Hajure (2020) Ethiopia Good quality	Cross‐sectional Questionnaire completed via interview	*N* = 310 (265 male, 45 female) Median age 30 years (IQR 10)	Total capacity 1111 at time of study Systematic random sampling Excluded seriously ill inmates (98.7% response rate of 314 invited) Sample size calculated	Standardised: PSQI (questionnaire translated to local language by independent reviewer) Reliability not stated Score ≥5 indicated poor sleepers	To assess the prevalence and associated factors of poor quality of sleep among prisoners Marital status, history of incarceration, residence, illiteracy and lifetime alcohol use had an impact on the prevalence of poor sleep quality	77.1% poor sleep quality (PSQI ≥ 5)

ADHD, attentional deficit hyperactivity disorder; CCSM, cross‐cutting symptoms measure; DBAS‐16, dysfunctional beliefs and attitudes about sleep; DSM‐5, Diagnostic and Statistical Manual of Mental Disorders 5th edition; ICD‐10, International Classification of Diseases version 10; ICSD‐3, International Classification of Sleep Disorders third edition; IQR, interquartile range; ISI, Insomnia Severity Index; MAPS, multivariable apnea prediction score; MEQ, Morningness–Eveningness Questionnaire; PIRS, Pittsburgh Insomnia Rating Scale; PSQI, Pittsburgh Sleep Quality Index; PTSD, post‐traumatic stress disorder; SCI, Sleep Condition Indicator; SD, standard deviation; SHI, Sleep Hygiene Index.

The 12 studies were published between 2014 and 2020. Sample sizes ranged from 95 to 1491, and participants were aged between 18 and 85 years. Response rates were reported by most studies (*n* = 9), and ranged from 18% to 99.5%. The samples had diverse countries of origin (i.e. Switzerland [*n* = 1], Taiwan [*n* = 1], UK [*n* = 2], India [*n* = 1], Turkey [*n* = 1], China [*n* = 1], Ethiopia [*n* = 2], Pakistan [*n* = 1], USA [*n* = 1] and France [*n* = 1]). Studies also included male and female participants: nine were solely, or predominantly, a male sample; one included a solely female sample; and two had a proportionate male to female sample. Most studies (*n* = 7) used voluntary sampling of the prison population (i.e. following screening of inclusion/exclusion criteria), three studies used random sampling, one study used consecutive sampling, and in one study the sampling method could not be established.

### 3.2 Sleep quality measure

All studies measured sleep quality through self‐report questionnaire. Seven studies utilised an intermediary to assist with the questionnaire completion. The most common method (*n* = 5) comprised interviewing the participant with the questionnaire; with other studies (*n* = 2) questionnaire completion was overseen by a literacy teacher and a data collector. Five studies had participants complete the questionnaire independently.

The majority of studies (*n* = 10) employed standardised measures. Five studies measured insomnia using standardised questionnaires; three used the ISI (Acar et al., 2019; Li & Lai, 2019; Vogler et al., 2014), one used the Sleep Condition Indicator (SCI; Dewa et al., 2017a) and one used the Pittsburgh Insomnia Rating Scale (Raha et al., 2018). Six studies utilised the PSQI to measure sleep quality (Abdu & Hajure, 2020; Acar et al., 2019; Barker et al., 2016; Dewa et al., 2017a; Getachew et al., 2020; Harner & Budescu, 2014). Barker et al. (2016) used the specific perceived sleep quality item from within the PSQI, where perception was a particular interest of the study. Other standardised measures were used to capture other various sleep factors including the Sleep Hygiene Index and the Morningness–Eveningness Questionnaire. The use of an unstandardised measure was less common, with only two studies using unstandardised measures as their only measure: Geng et al. (2020) designed questions based on DSM‐5 and International Classification of Sleep Disorders third edition (ICSD‐3) criteria for insomnia in order to measure prevalence of insomnia; and Goudard et al. (2017) designed questions to measure sleep satisfaction in their participants.

There were some discrepancies in how individual studies chose to interpret standardised measures. Studies using the ISI varied in the score thresholds used for determining insomnia: Li and Lai (2019) used a cut‐off score of ISI ≥ 9; Vogler et al. (2014) used ISI ≥ 10; and Acar et al. (2019) used ISI ≥ 15. This highlights how the detection of insomnia was more conservative in the latter than the former studies. However, it is interesting to note that Acar et al. (2019) reported the highest prevalence of insomnia in this subsection of studies, followed by Vogler et al. (2014), and then Li and Lai (2019). According to the measure, ISI ≥ 15 would distinguish “clinical insomnia”, whereas ≥10 and ≥9 are part way in the “subthreshold” range of scores of 8–14 (Morin et al., 2011).

There was further inconsistency in the use of PSQI score thresholds with Dewa et al. (2017a), Getachew et al. (2020), and Harner and Budescu (2014) using the suggested PSQI > 5 for distinguishing poor sleep. However, Abdu and Hajure (2020) and Acar et al. (2019) used PSQI ≥ 5. Barker et al. (2016) did not describe the threshold used for PSQI nor did they report the prevalence of poor sleep measured by the PSQI global score. This, again, means that Abdu and Hajure (2020) and Acar et al. (2019) overestimated the prevalence of poor sleep quality by including a score of 5 as poor‐ rather than good‐quality sleep (Backhaus et al., 2002).

Ishfaq and Kamal (2019) measured sleep problems (amongst other psychiatric symptomology) with the 23‐item DSM‐5 cross‐cutting symptoms measure (CCSM), and the prevalence of sleep problem was estimated from one question and should be evaluated with caution due to this crude measurement.

### 3.3 Prevalence

The quality of previous estimates of prevalence of insomnia and/or sleep problems in prison populations has often been hindered by the measurement being a byproduct of other study aims (Dewa et al., 2015). The main aim of eight of the studies included in the current review was to measure the prevalence of sleep problems in the prison setting, and four measured sleep factors in order to investigate correlates with other comorbidities (e.g. psychiatric disorders, anger and attentional deficit hyperactivity disorder [ADHD]).

#### 3.3.1 Insomnia prevalence

The first six rows of Table 1 show studies including reported prevalence of insomnia specifically. Estimated prevalence of insomnia varied, with rates ranging from 26.2% to 72.5%. Examination of the study characteristics in greater detail offers explanations as to the considerable heterogeneity in these prevalence rates. The sample sizes of the six studies reporting insomnia prevalence vary considerably (49–1491 participants). Interestingly, the two studies with the largest samples (1490 and 1491 participants; Geng et al., 2020; Li & Lai, 2019) report the lowest prevalence, suggesting a magnified prevalence in smaller studies. However, Vogler et al. (2014) had the smallest sample size (*n* = 49) and reported the third lowest prevalence rate (33%). Whilst five of these six studies have used standardised measurements, the differences in measurement and cut‐off for insomnia using ISI stated previously fundamentally undermine any meaningful comparison between the reported prevalence rates. Furthermore, Geng et al. (2020), who had one of the largest samples but smallest prevalence, used an unstandardised measure that although it was designed according to insomnia diagnostic criteria, has not been tested for its construct‐validity.

A further consideration relates to gender difference: two of the six studies included a representative female sample. When gender difference is taken into account, the male prevalence of insomnia is estimated to be between 26.2% and 65%, and the female prevalence of insomnia is estimated to be between 70.6% and 72.5%. This difference is stark, and although the comparison in gender prevalence is very limited due to a small comparison sample of females and possibility of over‐estimation, it does generate an important narrative in support of known gender differences in insomnia.

#### 3.3.2 Poor sleep quality prevalence

Table 1 shows nine studies reporting prevalence of poor sleep quality. Estimated prevalence of poor sleep quality varied, with reported rates ranging from 42.8% to 88.2%. Further examination offers explanations as to the heterogeneity in these prevalence rates. Poor sleep quality is perhaps a more common and subjective concept to measure than insomnia, so higher incidence and greater variation might be expected. Interestingly, the four studies that reported the lowest prevalence of poor sleep quality used unstandardised or very simplistic measures (Barker et al., 2016; Geng et al., 2020; Goudard et al., 2017; Ishfaq & Kamal, 2019). This suggests an underestimate of prevalence when compared with more reliable measures.

When the five studies utilising PSQI are considered, the same fundamental flaw remains with differing measurement cut‐offs, which undermines meaningful comparison. When we examine the prevalence according to the cut‐off used, however, the three studies using the recommended PSQI > 5 prevalence have a range from 62.5% to 88.2% (Dewa et al., 2017a; Getachew et al., 2020; Harner & Budescu, 2014); and the two using the over‐estimated PSQI ≥ 5 reported prevalence is actually within the above range at 70.4%–77.1% (Abdu & Hajure, 2020; Acar et al., 2019). Similarly to insomnia prevalence, samples sizes varied between studies and had predominantly male samples.

#### 3.3.3 Short sleep duration

Four studies reported short sleep duration (Barker et al., 2016; Geng et al., 2020; Goudard et al., 2017; Vogler et al., 2014). The reported prevalences of less than 6 hr of sleep a night were 17.4%–40%, and Goudard et al. (2017) reported approximately 35% had less than 5 hr sleep a night. Barker et al. (2016) reported that their findings supported the notion that sleep quality as opposed to sleep quantity is more important with regard to predicting aggressive behaviour.

#### 3.3.4 General population comparisons

Some studies commented on how their prevalence rates compared with the reporting country's general population estimate (Abdu & Hajure, 2020; Dewa et al., 2017a; Geng et al., 2020; Goudard et al., 2017; Li & Lai, 2019). All studies reported a higher prevalence of poor sleep in the prison population than the general population, with slightly differing prevalence rates between the countries. For example, estimates of insomnia prevalence are reported to be smaller in China (Cao et al., 2017; Geng et al., 2020) than in the UK (Dewa et al., 2017a; Espie et al., 2012). Variable measurement is seen as a significant problem in estimating insomnia prevalence (Cao et al., 2017; Dewa et al., 2015). Dewa et al. (2017a) was the only study to report the comparative general population estimate measured using the same insomnia questionnaire as their study. Table 2 displays an approximate comparison of prevalence of poor sleep factors in prison and general populations.

**TABLE 2:** Current review prevalence rates compared with general population estimates

	Prison prevalence rates of reviewed studies using standardised measures	General population estimates of prevalence
Insomnia prevalence	26.9%–72.5%	6%–30%^a^ ^,^ ^b^
Poor sleep quality prevalence	62.5%–88.2%	25%–36%^a^ ^,^ ^c^
< 6 hr of sleep duration per night	17.4%–40%	11%–29.9%^d^ ^,^ ^e^

^a^ Morin and Benca (2012).

^b^ Roth (2007).

^c^ Hinz et al. (2017).

^d^ Bin et al. (2013).

^e^ Luckhaupt et al. (2010).

#### 3.3.5 Comorbidities

Some studies measured correlates of poor sleep including aggression, drug or alcohol use, anxiety, depression, suicidality, post‐traumatic stress disorder (PTSD), ADHD and physical health. Barker et al. (2016) reported an association between poor sleep and higher aggression, and Vogler et al. (2014) also reported this association with aggression with the additional correlate of higher ADHD symptomology with poor sleep. Abdu and Hajure (2020), Dewa et al. (2017a) and Getachew et al. (2020) found associations between certain drug or alcohol abuse histories and poor sleep quality. Li and Lai (2019) reported that anxiety was an independent predictor of insomnia. Dewa et al. (2017a) reported that those prisoners with insomnia were more likely to report symptoms of anxiety, depression and suicidality. Acar et al. (2019) and Harner and Budescu (2014) found that insomnia and poor sleep quality were higher in those with PTSD. Dewa et al. (2017a), Geng et al. (2020) and Li and Lai (2019) reported positive correlations between insomnia/poor sleep quality and poor physical health status.

Some studies measured and reported on other demographic correlates as well as prison environmental factors. Demographic correlates included a negative correlation between sleep duration and older age (Geng et al., 2020), a negative correlation with poor sleep quality and education level (Abdu & Hajure, 2020), a positive correlation between insomnia and having religious beliefs (Li & Lai, 2019), a positive correlation between poor sleep quality and previous criminal activity or incarceration (Abdu & Hajure, 2020; Ishfaq & Kamal, 2019), and a higher insomnia prevalence in those who were divorced or widowed (Geng et al., 2020).

Dewa et al. (2017a) not only alluded to environmental factors impacting sleep within prison, as other studies did, but they also measured it within their study, developing a Prison Environment Sleep Questionnaire. They reported that those with insomnia had significantly higher reports of environmental disturbances including noise, temperature, light and mattress discomfort. Similarly, Goudard et al. (2017) also measured environmental factors within their questionnaire, with 66% of poor sleepers (and 37% of good sleepers) complaining of noise pollution, and 40% of poor sleepers (and 21% of good sleepers) reporting discomfort with temperature. Harner and Budescu (2014) took qualitative data from the PSQI to report on additional factors affecting sleep: 40% of their female sample disclosed problems including environmental noise and bed discomfort being a problem three or more times a week. Additionally, these three studies also referenced ruminating thoughts affecting sleep for a large proportion of participants, likely to be associated with poor mental health.

## 4 DISCUSSION

The current systematic review identified 12 cross‐sectional studies reporting on prevalence of insomnia or poor sleep quality using self‐report measures within adult prisoners. Results showed that research measuring sleep quality within prisons has increased over recent years, and that the estimated prevalence rates of sleep problems in prisons is variable. Whilst improvement in the consistency of measurement of sleep factors through increased use of standardised tools was shown, there remains discrepancy in the parameters used within measurement tools (e.g. ISI and PSQI). This critically undermines comparison of estimated prevalence rates and needs to be consistently agreed for the field to progress. Furthermore, the studies varied methodologically on quality, sampling (size and method) and administration of self‐report questionnaires, which probably also impacted on the variability of prevalence rates.

Within six reviewed studies measuring insomnia, prevalence rates ranged from 26.2% to 72.5%. There was a gender difference observed as two of the six studies had a representative female sample: females accounted for the highest prevalence rates of insomnia (70.6%–72.5%), whereas male (or predominantly male) prevalence rates were 26.2% to 65%. There was discrepancy in the measurement used, the cut‐off to determine insomnia (on the ISI) and the sample size, which caused concern for the credibility of the rates.

Within the nine studies measuring poor sleep quality, prevalence rates ranged from 42.8% to 88.2%. There was some variability in measurement used, which raised questions on reliability. Five studies used PSQI to report the prevalence of poor sleep with rates between 62.5% and 88.2%. However, there again was variability in the cut‐off used to determine poor sleep with the possibility of over‐estimating the prevalence of poor sleep for two studies. Conversely, the prevalence rates did not reflect this, but this incongruity does call to question the accuracy of the reported prevalence rates. Four studies also recorded and reported on short sleep duration (< 6 hr sleep per night), with prevalence rates between 17.4% and 40%. All prison population sleep‐related prevalence rates were higher than general population estimates (Morin & Benca, 2012).

Dewa et al.’s (2015) previous integrative review reported a varied prevalence of insomnia within prison populations with rates of 10.9%–81% from 12 studies between 1974 and 2012. However, none of the reviewed studies used a recommended standardised measure to assess insomnia by diagnostic criteria (e.g. clinical interview, ISI or SCI). Therefore, the current review offers confidence that the prevalence rate (of between 26.2% and 72.5%) is likely to be more accurate.

Elger's (2007) scoping review concluded that insomnia in prisons was more likely to be a primary problem rather than a secondary one to other conditions. Indeed, Goudard et al. (2017) reported in their study that 57% of poor sleepers' difficulties had begun in prison, whilst 31% already had sleep problems that became worse in prison, suggesting a variety of possible causes and interactions. Although not a specific aim of this review, comorbidities of sleep problems have been noted within the reviewed studies. Studies included many correlates of sleep problems including mental health condition, physical health and demographics. The incidence of psychiatric disorder within prison populations is known to be high (Fazel & Danesh, 2002) and it is likely to impact wellbeing, including sleep quality. However, some of the studies in this review excluded participants based on their psychiatric diagnosis (Geng et al., 2020; Getachew et al., 2020; Raha et al., 2018). This may have implications for the validity of reported prevalence rates of sleep problems, with a probable underestimation. Clearly the relationship between sleep and mental health is a complex one, with particular difficulties in measurement and in determining cause and effect. The importance of sleep, however, within the picture of mental health is vital given the well‐established association between insomnia, depression and suicide (Dewa et al., 2017a).

None of the studies in this review used recommended objective sleep measures, such as actigraphy or polysomnography: this was seemingly due to the barriers of using such measures within prison settings (Barker et al., 2016). The use of self‐report does cause some doubts in regard to subjectivity: self‐report sleep measures do not always correspond with objective ones (Girschik et al., 2012). However, more than half of the studies (*n* = 7) utilised a mediator to assist with questionnaire completion. This method reduces the risk of misunderstanding, data omission, and can also increase uptake due to rapport building when conducting research in prison settings (Sutton, 2011).

Studies included within this review represent a variety of countries and these differences must be considered. Cultural difference may explain some variance in prevalence of insomnia/poor sleep quality. It has been reported that the prevalence of insomnia is lower in China and other Asian countries when compared with Western countries (Cao et al., 2017; Zhang & Wing, 2006). These differences could be attributable to a myriad of factors including lifestyle, values and socioeconomics. Younger adults appear to report higher levels of insomnia in China, whereas the trend is for insomnia to more commonly be associated with older age in other populations. Again, this could be attributable to many things, for instance the use of technology and its negative effect on sleep hygiene (Cao et al., 2017).

Differences in prison settings between countries must also be considered, particularly with regard to environmental factors. Comment on specific differences is outside the scope and purpose of this review; however, generally, there will be variation in prison conditions relating to the criminal justice system, level of security and practices, prison capacity and facilities. Such differences will likely have a direct impact on the opportunity for quality sleep (Abdu & Hajure, 2020). Included studies reported on external environmental factors impacting on sleep, such as overcrowding, prison officer rounds, bed comfort, temperature, light and noise (Dewa et al., 2017a; Goudard et al., 2017; Harner & Budescu, 2014). The internal cognitive and emotional process of incarceration was mentioned in some studies and its impact on sleep, for instance the feelings of guilt, rumination, anxiety, fear of violence and isolation (Getachew et al., 2020; Harner & Budescu, 2014; Raha et al., 2018). Such findings on internal and external factors affecting sleep can offer insights for interventions for improving sleep quality such as Cognitive Behaviour Therapy for Insomnia (Dewa et al., 2017b) and more general environmental solutions like earplugs (Goudard et al., 2017).

In addition to the methodological considerations already discussed, there are several further limitations of the included studies. Given the nature of the review, all studies were cross‐sectional and cannot support conclusions on causality. The sample sizes varied considerably, with some reporting very small samples, which is problematic when inferring prevalence. Sampling methods also varied: some studies reported response rates that were overall quite positive; however, largely opt‐in sampling methods may have created some bias alongside the differing exclusion criteria between studies. Generalisability of the results will be difficult given the specific parameters of the study question (i.e. the results are limited to each prison the study was conducted in and the samples are heavily weighted towards males, as is the case throughout prison populations). This raises further caution to generalisability of gender differences. It must also be clarified that all of the studies collected their data prior to the COVID‐19 pandemic. The impacts of pandemic restrictions on a prisoner's sleep is an area of research in need of further exploration.

There are some limitations to this review. There was a relatively small number of studies included, and these were split between reporting the prevalence of insomnia versus poor sleep quality. The exclusion criteria used in this review narrowed the opportunity to review more studies (i.e. non‐English articles, abstracts). The small publication timeframe of studies searched also impacted on the identified number of studies reviewed. Heterogeneity of these studies, alongside the small number measuring and reporting on insomnia specifically, prevented a meta‐analysis. Nevertheless, the current review has demonstrated an improvement in recent years in regards to research activity and study quality: the result is that we can be more confident that there is a slightly narrower range of prevalence estimates.

### 4.1 Future research recommendations

- Measure sleep quality in prisoners using the recommended parameters of validated self‐report measures (e.g. ISI and PSQI).
- Use objective measures, for example actigraphy and polysomnography.
- Study prisoners’ sleep quality longitudinally to include pre/post prison stay. This will help explore prison‐specific factors impacting sleep.
- Provide transparency on prison environmental factors within future studies to help clarify the impact of these on sleep quality.
- Independently study male and female differences of sleep quality/insomnia as this difference appears to be meaningful and particularly pronounced in prison populations (Dewa et al., 2017a; Harner & Budescu, 2014; Raha et al., 2018).

These considerations would aid the understanding of the aetiology of insomnia, and help in the development and priority afforded to interventions in prisons.

## 5 CONCLUSION

The prevalence of poor sleep quality and insomnia within the prison population is variable and above the general population average. There are a myriad of known factors influencing sleep problems including mental health difficulties, poor physical health and prison environmental factors. This review has highlighted that research over the past decade has increased in measuring the sleep of prisoners, with a rise in utilising validated measures. This is an important step due to the negative emotional and physical implications of inadequate sleep quality and quantity. Future epidemiological studies should seek to measure insomnia in accordance with diagnostic criteria, objectively and longitudinally where possible, and/or use recommended and consistent parameters on validated self‐report measures. Ultimately, the high prevalence of substandard sleep within prisons should be recognised as a priority to screen and treat routinely.

## AUTHOR CONTRIBUTIONS

Supervised and inter‐rater reviewed by Dr Lee Hogan in partial fulfilment of Doctorate in Clinical Psychology.

## CONFLICT OF INTEREST

None.

## Data Availability

The data that support the findings of this study are available from the corresponding author upon reasonable request.
